# Two of a Kind? Similarities and Differences between Runners and Walkers in Sociodemographic Characteristics, Sports Related Characteristics and Wearable Usage

**DOI:** 10.3390/ijerph19159284

**Published:** 2022-07-29

**Authors:** Kobe Helsen, Mark Janssen, Steven Vos, Jeroen Scheerder

**Affiliations:** 1 Policy in Sports & Physical Activity Research Group, University of Leuven, 3001 Leuven, Belgium; kobe.helsen@kuleuven.be; 2 School of Sport Studies, Fontys University of Applied Science, 5644 HZ Eindhoven, The Netherlands; mark.janssen@fontys.nl (M.J.); steven.vos@fontys.nl (S.V.); 3 Department of Industrial Design, Eindhoven University of Technology, 5612 AZ Eindhoven, The Netherlands; 4 Flemish Policy Research Centre on Sports, 3001 Leuven, Belgium

**Keywords:** mobile application, sports watch, smartwatch, online survey, COVID-19, attitude, motivation, digitalisation, profile, UTAUT2

## Abstract

As the two prime examples of sport light, running and walking have become very popular sports activities in the past decades. There are references in the literature of similarities between both sports, however these parallels have never been studied. In addition, the current digitalisation of society can have important influences on the further diversification of profiles. Data of a large-scale population survey among runners and walkers (*n* = 4913) in Flanders (Belgium) were used to study their sociodemographic, sports related and attitudinal characteristics, and wearable usage. The results showed that walkers are more often female, older, lower educated, and less often use wearables. To predict wearable usage, sports-related and attitudinal characteristics are important among runners but not among walkers. Motivational variables to use wearables are important to predict wearable usage among both runners and walkers. Additionally, whether or not the runner or walker registers the heart rate is the most important predictor. The present study highlights similarities and differences between runners and walkers. By adding attitudinal characteristics and including walkers this article provides new insights to the literature, which can be used by policymakers and professionals in the field of sport, exercise and health, and technology developers to shape their services accordingly.

## 1. Introduction

From the 1960s onwards, the number of sports participants has increased over the years in different European Union (EU) countries [1]. At present, there are indications that the number of sports participants is stagnating or decreasing [2]. Sport can be practiced in a variety of contexts, and while the more traditional sports clubs are losing their share of general sports participation [3], there are other settings that have been gaining popularity in recent years (such as participation in fitness centres) [4]. In addition, people are becoming increasingly active in non-organised contexts as well (so-called sport light; e.g., alone, unorganised with friends, in participatory sports events, etc.) [5,6].

Running and walking are the two prime examples of this evolution towards sport light and are rated as two of the most popular sports in different EU countries [1,5,7], as well as in the United States and Canada [8,9]. Traditionally, running was practiced by (competitive) athletes in track and field clubs or as part of school curricula [5,10]. In the past decades, however, the profile of runners has become more heterogenous (e.g., sex, age, motivations to run) [11,12,13]. Several studies have been undertaken to analyze the profiles of runners [14,15], often in the context of event participation [11,12,16,17,18,19]. Nonetheless, it is assumed that the profile of event runners differs from runners in general [18]. Past research highlighted the need for and utility of attitudinal variables, in addition to demographic characteristics, to distinguish different types of sports participants [17,19,20,21,22,23].

Although running has been extensively researched, knowledge on the popularity of walking as well as of the profile of walking participants is limited. Past research has mainly focused on the profile of ultra-endurance walkers [24], on the influence of walking on individuals in disease related settings [25,26], or on the consequences of applying intervention walking programs [27,28,29]. Additionally, defining walking seemed to be difficult in past research, as there is no uniform term to capture (different forms of) walking [30,31,32,33], as short walks (i.e., less than thirty minutes) are not regarded as walking activities [34,35], or as some participation studies showed overall participation numbers including and excluding walking [30,36].

As a result of the COVID-19 pandemic, changes have occurred in how people practice and experience active sports participation [37], which could lead to a diversification of sports participants’ profiles [38]. Many countries implemented (strict) measures to combat the spread of the virus [39,40]. Belgium, which serves as a context for this study, imposed a so-called light lockdown. This light lockdown meant a closing of schools, sports clubs, sports infrastructures, and the cancellation of all events, but people were encouraged to exercise outdoors (limited, however, to walking, running, cycling, and roller skating) with one sporting friend [18,41,42]. Because a large share of sports participants were not able to practice their favourite sport in sports infrastructures or sports clubs, people discovered other easy-to-practice sports, such as running, walking or cycling (low threshold, no infrastructure, limited cost, possible to practice alone, etc.) [38,43,44]. In addition, both sports fit the current trend towards escapism and simultaneously staying connected through screens and social media [45,46].

In the current technological society, the sports sector was introduced by smart technologies and devices (e.g., online subscriptions to participate in running events, sensors to track your running cadence or length of your steps, use of mobile apps, etc.) [47]. Currently, the majority of sports participants use wearables [14], as research shows that 75 to 90 percent of event runners use applications or sports watches [11,20,48]. Depending on the type of runner, however, other kinds of technology are used. Both sociodemographic and sports related variables turned out to be decisive for the technology used [11,14,20,48,49]. A commonly used theory to explain the use of technology is the Unified Theory of Acceptance and Use of Technology (UTAUT), which was developed by Venkatesh et al. [50]. The model proposes four factors or motivations that predict the intention to use technology (i.e., performance expectancy, effort expectancy, social influence, and facilitating conditions). It was further validated and extended (UTAUT2) by Venkatesh et al. [51] in a consumer context by adding three constructs or motivations to improve the variance explained by the model (i.e., hedonic motivation, price value, experience and habit). The use of the model has vastly increased in a multitude of settings [52].

It is expected that COVID-19 was a strong accelerator for the wearable usage of runners for numerous reasons. Some running event organisers transformed their events to virtual events. Therefore, event participants needed to have smart watches, GPS-tracking devices, or apps to track and share performances [53]. In addition, due to the disappearance of club organised sport because of COVID-19 measures, club organised sports participants tried to stay connected with teammates by (online) sharing their workouts. After all, there was an exponential growth of online tutorials and eSports during the pandemic [49,54]. Finally, studies executed since the COVID-19 measures indicated that approximately one in five sports participants used digital media (or were interested to use it) for sports activities [18,49].

Therefore, this study aims to analyse the similarities and differences between running and walking participants concerning their (sports) profile, attitudinal variables and wearable usage. Second, this paper further examines the wearable usage of running and walking participants by investigating the predictive factors of using smartphone applications or sports watches.

## 2. Materials and Methods

### 2.1. Study Design and Procedure

An online questionnaire, the Leuven Running & Walking Survey 2.0 (LRWS2.0), was used to collect data among Flemish running and walking participants of eighteen years and older. By implementing a cross-sectional study design, data were collected between 20 November 2021 and 20 December 2021 by means of Qualtrics software.

The link to the online questionnaire was widely disseminated by different partners: (i) the Flemish athletics federation and the Flemish walking federation; (ii) other Flemish sports federations that offer running and/or walking (i.e., sports federations that offer multiple sports); (iii) running and/or walking event organisers (e.g., commercial and non-commercial); and (iv) the personal networks (e.g., academic and non-academic) of the authors. First, information on the background and purpose of the study was provided. Next, participants had to give their consent to treat data anonymously in the context of the research. The study was conducted in accordance with the Declaration of Helsinki.

Important characteristics to define sports participants include (i) sports participation in free time in the twelve months before the survey, with intentional engagement and adapted clothing (also during holidays, but not during physical education classes at school; (ii) physical activities such as cycling to work, gardening or walking to the bakery are not included.’ [55] (p. 11). The authors of this study have extended the definition of sport to a broad range of activities (including running or walking) and set a number of conditions to be categorised as sport (such as intentional engagement or adapted clothing). The quite broad definition of sport that is used in the current study ensures that high participation numbers are shown but ensures applicability to the activity of walking too.

The widely deployed sampling strategy resulted in a total response of 10,316 participants. Respondents that did not complete the informed consent, were not running or walking participants, were not residents of Flanders and were under 18 years old were deleted from the dataset. After checking for missing data, the collected data were controlled for sex, age, and educational level by using population statistics to ascertain a representative response [56]. In that way, this paper refers to 4913 running (*n* = 2146) and walking (*n* = 2767) participants that run/walk at least one time per week.

### 2.2. Respondents

In the total sample, running participants are underrepresented (43.6%) compared to walking participants (56.3%). More than half of running participants are male (51.8%), between 35 and 54 years old (58.2%), and higher educated (57.0%). Walking participants are more likely to be female (55.0%), 55 years old and older (60.5%) and lower educated (63.6%).

### 2.3. Questionnaire

The questionnaire consisted of four parts, including (i) sociodemographic variables; (ii) sports related variables; (iii) attitudinal variables; and (iv) wearable usage variables. The largest part of the questionnaire was validated in past research on Flemish runners [15] and Dutch event runners [11,20].

*Sociodemographic variables* included (i) sex (male/female/other); (ii) age (birth year); and (iii) highest level of education achieved (primary education or no education/secondary education/higher education).

*Sports related variables* included (i) frequency of running or walking in the twelve months before the survey (times per week; running for running participants; walking for walking participants) [11,15,20]; (ii) main sport (perceived by respondent) in the twelve months before the survey (running or walking/other) [11,15,20]; and (iii) event participation as participant (never/at least once; running events for running participants; walking events for walking participants) [11,15,20].

*Attitudinal variables* were measured using a (sports) leisure involvement scale consisting of thirteen items and three constructs (considering running for running participants; considering walking for walking participants). The scale was developed by Laurent & Kapferer [57] and Zaichkowsky [58], and further elaborated and validated by Kyle et al. [59] and Helsen et al. [18]. The authors enriched the scale by adding one additional item from Kyle et al. [32]. Confirmatory factor analyses (CFAs) were used to verify the factor structure and reliability of this leisure involvement scale (by using AMOS 28). Two CFAs were executed (one for running participants and one for walking participants) as (1) the running and walking participants were not proportionally represented in the total sample, and (2) the running and walking participants could reveal different results. The initial models with three constructs and thirteen items achieved an unacceptable fit. Therefore, three items were deleted from the models (one item from each construct with factor loading below 0.6; [60]). The final models with three constructs and ten items had a good fit with acceptable RMSEA values (Table 1; CFI = 0.976, TLI = 0.959, RMSEA = 0.059 for running participants; CFI = 0.964, TLI = 0.938, RMSEA = 0.075 for walking participants; [61,62]). Reliability and convergent validity were achieved (CR values and AVE values met recommended levels; [63]). Discriminant validity was achieved, as squared correlations between constructs did not exceed AVE values [63].

*Wearable usage variables* included (i) whether the respondent used a wearable during running or walking (running for running participants; walking for walking participants) [11,20]; (ii) type of wearable that is used during running or walking participation (application for smartphone/(sports)watch or smartwatch/activity tracker/handheld GPS) [11,20]; (iii) which parameters are registered with the wearable (running time/distance/speed/heart rate/cadence/route; for the wearable that was used most often by the respondent) [11]; (iv) the purpose of the collected data of the wearable (nothing/look at data after training/monitor progress/adjust workouts; for the wearable that was used most often by the respondent) [11]; (v) motivation to use the wearable (for the wearable that was used most often by the respondent), measured using the a scale with eight constructs and 27 items [64] by using a five-point Likert scale ranging from strongly disagree to strongly agree. The scale was developed and tested on internal consistency by Fontys University of Applied Sciences and Eindhoven University of Technology. The scale is based on the Unified Theory of Acceptance and Use of Technology 2 (UTAUT2) model (which was developed in a general consumer context) [51] and translated to a sports context (i.e., applications and sports watches). Exploratory factor analyses (EFAs; principal components with varimax rotation) were used to verify the factor structure and reliability of the scale to measure motivations to use wearables as the scale has not yet been validated in past research. Four EFAs were executed (one for the application among running participants, one for the sports watch among running participants, one for the application among walking participants, and one for the sports watch among walking participants) as (1) the running and walking participants were not proportionally represented in the total sample, (2) the users of applications and sports watches were not proportionally represented in the total sample, and (3) the EFAs for running and walking participants, as well as for the users of applications and sports watches, could reveal different results. The initial results of the four EFAs are presented in Table S1 (added as Supplementary Material). Based on the data and considering the cut-off Eigenvalue of one, six, six, five and five constructs could be observed among the use of applications among running participants, the use of applications among walking participants, the use of sports watches among running participants, and the use of sports watches among walking participants, respectively [65]. Because of low and double factor loadings among the use of applications among running participants for ID3, and a low factor loading for the other three EFAs, this item was deleted. SI4 and SI5 are retained because of clear higher values on a certain construct. Despite a factor loading below 0.6, FC1 is retained for further analyses as it is loading on only one construct in all EFAs. Despite some double loadings of 0.3 or higher among the PV or FC items, all the items are kept in the analyses because of clear higher values on a certain construct. PE2 and PE6 are deleted because of low and double factor loadings. Despite low and double factor loading for HE1 among walking participants, the item is retained for further analyses as the item loadings are clear for running participants. Despite low and double factor loadings for some items of EE and HA, all items are retained for further analyses as the loadings are clear for running participants. Table 2 presents the final results of the EFAs (considering that ID3, PE2 and PE6 are deleted). The five-factorial models explain 60.153%, 61.505%, 57.999%, and 62.186% of the variance for the use of applications among running participants, the use of applications among walking participants, the use of sports watches among running participants, and the use of sports watches among walking participants, respectively. Most constructs obtain Cronbach’s Alpha values of 0.70 or higher, denoting constructs of acceptable reliability (Table 3; [66,67]). Four constructs obtain Cronbach’s Alpha values between 0.60 and 0.70 and two constructs obtain Cronbach’s Alpha values below 0.6 (denoting poor to moderate reliability; [62]). Despite the lower reliability (<0.6) of two constructs, the factor structure of Table 2 is kept as the scale has not yet been validated in past research.

### 2.4. Analyses

Differences between running and walking participants are analysed by using chi-square tests with Bonferroni adjustments. Furthermore, the predictive factors of using smartphone applications or sports watches are analysed by using two binary logistic regression analyses (one for running participants and one for walking participants; SPSS 28). These two wearable types (i.e., smartphone applications and sports watches) were selected as the sample size was only large enough for these types. For each binary logistic regression analysis, six models are used sequentially. The sociodemographic variables comprise the first model, the sports related variables comprise the second model, and the last four models include the wearable related characteristics (i.e., number of wearables used, motivation to use the wearable, which parameters are registered with the wearable, and purpose of collected data). These wearable related characteristics are added separately, allowing for the specific identification of the most determining factors.

## 3. Results

### 3.1. Profile of Running and Walking Participants

Running participants are active, as more than 50 percent (53.5%) run three to four times per week (Table 4). However, the share of walking participants who walk five times per week or more is significantly larger than the share of running participants who run five times per week or more. Only a quarter of running and walking participants have another sport other than running and walking as their main sport. Finally, running participants (91.4%) use a wearable more often compared to walking participants (59.5%). Most running participants use a (sports)watch/smartwatch, whereas an application for the smartphone is the most important wearable for walking participants. No significant differences can be found regarding the number of wearables that is used. Almost seven in ten running and walking participants use one wearable.

### 3.2. Predicting Wearable Types

Table 5 presents the binary regression analysis (six models) for the most important wearable among running participants (application or sports watch). The variance explained in the total model, when adding all variables, equalled 59.2%. This model had a good fit (χ² (27) = 875.502; *p* < 0.001). Sociodemographic variables did not contribute significantly to the differences in the dependent variable (i.e., having an application or sports watch as most important wearable). Sports related variables and attitudinal variables were important, as 13.3 percent of the variance in the dependent variable could be attributed to these variables. More specifically, running participants that ran more than three times per week, and running participants that participated at least once in a running event were more likely to have a sports watch as their most important wearable (compared to those who ran two times per week or less and those that never participated in a running event). Additional important contributing variables were the motivation to use the wearable (particularly social influence and habit with a negative and positive effect) and registering the heart rate with the wearable. Those who registered their heart rate were much more likely to have the sports watch as their most important wearable compared to those who did not register their heart rate.

Table 6 presents the binary regression analysis (six models) for the most important wearable among walking participants (application or sports watch). The variance explained in the total model, when adding all variables, equalled 58.1%. This model had a good fit (χ² (27) = 791.099; *p* < 0.001). Sociodemographic, sports related, and attitudinal variables did not contribute much to the differences in the dependent variable, with having an application or sports watch being the most important wearable. The number of wearables that was used accounted for five percent of the variance of the dependent variable. Walking participants that used more than one wearable were more likely to have the sports watch as the most important wearable, compared to those who use only one wearable. The motivations to use the most important wearable were important as well, as 13.8 percent of the variance in the dependent variable could be attributed to these five motivational factors. More specifically, the motivations ‘price and support values’ and ‘habit’ had a positive significant influence, whereas ‘social influence’ had a negative significant influence on the dependent variable. Lastly, those who registered their heart rate were much more likely to have the sports watch as the most important wearable compared to those who did not register their heart rate.

When comparing the results of running and walking participants in Table 5 and Table 6, the explained variance of both final models are very similar (59.2% for runners and 58.1% for walkers). However, differences in predictive variables could be observed. The sports related variables and attitudinal variables were more important explanatory variables among running participants compared to walking participants (13.3% vs. 2.4%). Conversely, the number of wearables that was used by participants was a more important explanatory variable among walking participants (explaining 5.7% of the variance) than among running participants (explaining 0.2% of the variance). In addition, this variable had a positive influence among walking participants, but a negative influence among running participants. Finally, registering the heart rate with the wearable was a strong predictor among both running and walking participants.

## 4. Discussion

The profile of running participants got more diverse in past decades [5,11,12,13]. In addition, knowledge on the profiles of walking participants in general is sparse. Earlier studies have shown that profiles of running and walking participants further diversified because of COVID-19 [18,37,38,41,42,44]. Due to the temporary closing of sports clubs and sports infrastructure, some sports participants discovered the ease of running and walking (low threshold, low cost, etc.) [38,43,44] as they were two of the few sports that were allowed to be practiced in Belgium [18,41,42]. In addition, it is expected that COVID-19 caused an increase in the use of wearables because of the need of these technologies for virtual sports events or to stay connected with friends during lockdowns. This, in turn, led to a further diversification of profiles of running and walking participants. By considering and combining the two prime examples of sport light (i.e., running and walking) in this study, a reference point is provided which will facilitate the interpretation of results.

Running participants are more often male, younger, and higher educated compared to walking participants. In general, more running participants are frequently sports active (at least three times per week) and use a wearable when participating in running compared to walking participants. Past research showed that runners frequently used wearable technology, such as (sports)watches/smart watches and smartphone applications [14,20,23,48,68]. Approximately 50 to 60 percent of participants in the 2014 and 2015 half marathon of Eindhoven (The Netherlands) used a sports watch and roughly 35 to 55 percent used a smartphone application [20,23]. Similar results were found in other research, with 44 percent of Dutch runners using a smartphone application while running [69]. In addition, Clermont and colleagues [14] concluded that around three quarters of Canadian residents used a GPS running watch, more than 50 percent used a smartphone application and more than twenty percent used a smart watch. Compared to past research, it seems that Flemish runners more often use wearable technology. Stragier and colleagues [68] found that 57 percent of runners used an online fitness community, which could be compared with smartphone applications. The relatively high use of smartphone applications from Stragier and colleagues [68], however, cannot be confirmed in this study. This could be due to the increasingly intertwining use of applications and sports watches by sports participants in the past years. Forty percent of runners used an application while running and twenty percent considered the application as the most important type of wearable. The current study did not make, like Clermont and colleagues [14], a distinction between sports watches or smart watches, but combined them in one category ‘(sports)watch/smartwatch’. Combining both wearables may explain the relatively high numbers that are found in this study. Almost 85 percent of runners indicated that they had used a (sports)watch/smart watch and 80 percent identified this wearable as the most important one. Combining these two wearables can explain this elevated percentage, but the influence of COVID-19 is not negligible either.

Previous research highlighted that experienced runners more often use sports watches instead of smartphone applications compared to novice or inexperienced runners [11,14,23]. It is not surprising that walking participants more often use a smartphone application and less often use a (sports)watch/smartwatch compared to running participants as the psychological needs and stress to which the body is subjected (e.g., frequency and intensity) are different for both sports. Furthermore, walking participants are more interested in walking predetermined routes and are less interested in registering their heart rate or speed. Logically, their most popular wearable is the application because it is an easy and inexpensive way to track routes.

Research on explanatory variables for the use of wearables is sparse. Janssen and colleagues [20] concluded that, among event runners, the older running participants, those who participated in a club organised setting and those who participated in more than one running event per year, were less likely to use running apps. In addition, the authors found that older running participants, those who trained at least twice a week, those who participated in a club organised setting, and those who participated in more than one running event per year were more likely to use sports watches. Clermont and colleagues [14] also found that frequent runners prefer sports watches over mobile applications as well. To gain a deeper understanding of sports participants, it is important to grasp attitudinal variables and opinions [11,19,21], which is addressed in the current study. In line with Janssen et al. [20], this study demonstrated that frequent runners and event runners are more likely to use (sports)watches/smart watches and less likely to use applications. However, unlike Janssen et al. [20], no influence of sociodemographic variables was found. In addition, and in line with Janssen et al. [11], attitudinal variables showed to be important to predict wearable usage as the running involvement variables and motivational variables to use wearables were significant predictors explaining a large variance in wearable usage. This study suggests that highly involved runners are more likely to use sports watches over smartphone applications, which is an interesting avenue for wearable developers in targeting consumer groups. Additionally, if running participants experience the use of a wearable as a habit, they are more likely to use sports watches as well. This could possibly be due to the fact that sports watches are not only used for the sports activity itself, but have become part of everyday life (compared to smartphone applications). In the literature, the knowledge on the use of wearables among walking participants is absent. Compared to running participants, the sports related variables (frequency, event participation, involvement with walking) were of less to no relevance in predicting the wearable usage among walking participants. In addition, no differences were found in wearable usage between high and low involved walking participants. However, for this group of participants, the motivational variables to use wearables were significant predictors as well. It appeared that walking participants who give great value to support are more likely to use a sports watch. COVID-19 has had a strong influence on how sports are practiced and experienced [38,43,44]. It is expected that the digital field of applications and devices will evolve significantly in the near future given the short-term changes and achievements since COVID-19. Lastly, this article makes two final important contributions to the literature. First, Janssen and colleagues [11] indicated that event runners who use apps less often register heart rate data compared to event runners who use sports watches. The current article shows that whether or not the sports participant is registering their heart rate seemed to be the most important and distinctive variable in predicting the use of applications on the one hand or (sports)watches/smartwatches on the other hand, both among running and walking participants. Second, the number of wearables that is used has an opposite effect among running and walking participants. A running participant that uses more than one wearable is less likely to have the (sports)watch/smartwatch as most important wearable. A walking participant, however, that uses more than one wearable is more likely to have the (sports) watch/smartwatch as the most important wearable.

### Limitations and Future Research

The current study has some limitations. To collect data, preference was given to a quantitative approach by administering a large-scale population survey. Although this sampling method stimulates self-selection bias, this method was chosen over others. In order to overcome this methodological issue, the data were weighed by using population statistics to establish a representative response [56]. Second, the scale to measure the motivation to use wearables was reliable for the largest part, with some shortcomings. The scale is an optimisation of the scale developed by Venkatesh et al. [51] by making the translation to a sports context. The final scale was deliberately kept quite broad in this article, despite the few low factor loadings and reliability values, by removing only three items because it had not been validated in past research. Third, the scale to measure the motivation to use wearables was developed in the context of running participants. However, in this article it was used for walking participants as well without any adjustments. Next, the different options of types of wearables were limited to four (i.e., application, (sports)watch/smartwatch, activity tracker, handheld GPS). There are more types, but the authors reasoned that most types can be categorised into one of these four. Lastly, to limit the length of the questionnaire, some questions (i.e., what is registered, the purpose of registered data, motivation) were only asked for the most important wearable. If a respondent used more than one wearable, they had to identify the wearable which they used most.

Future research can supplement current knowledge by further validating the scales that are used in this study, more specifically the scale to measure the motivation to use a wearable. In addition, the study can be reproduced in other countries to test whether a similar use of technology while running or walking is apparent. Further, an extension to other sports that can typically be practiced in unorganised settings (e.g., cycling, triathlon) is encouraged.

## 5. Conclusions

In general, running participants more often use (sports)watches/smartwatches compared to applications while running, while the opposite is true for walking participants. The motivation to use wearables is an important factor to predict the type of wearable that is used most often for both running and walking participants. In particular, if it is more of a habit to use a wearable while being active, both running and walking participants are more likely to use a sports watch. On the other hand, if participants are more prone to the social influence of others, they are less likely to use a sports watch. In addition, many sports participants track their running time, covered distance and speed while running or walking. The tracking of these parameters are not decisive in determining the wearable that is used. However, when participants want to monitor their heart rate, they need a device that is suitable for that purpose (e.g., a sports watch).

## Figures and Tables

**Table 1:** Confirmatory factor analysis of involvement in running/walking.

Variable	Running Participants CFI = 0.976; TLI = 0.959; RMSEA = 0.059				Walking Participants CFI = 0.964; TLI = 0.938; RMSEA = 0.075			
	β	AVE	CR	SC	β	AVE	CR	SC
**Attraction**		0.59	0.85	0.23–0.50		0.62	0.86	0.17–0.43
[sport] ^1^ is important to me.	0.74				0.71			
Participating in [sport] ^1^ is one of the most enjoyable things that I do.	0.89				0.91			
Participating in [sport] ^1^ is one of the most satisfying things that I do.	0.80				0.89			
I have little or no interest in [sport] ^1^.	0.62				0.60			
*[sport] ^1^ offers me relaxation when pressures build up.*								
**Centrality**		0.62	0.82	0.40–0.50		0.59	0.81	0.42–0.43
I find a lot of my life is organised around [sport] ^1^.	0.84				0.83			
[sport] ^1^ plays a central role in my life.	0.88				0.85			
I enjoy discussing [sport] ^1^ with my friends.	0.62				0.61			
*Most of my friends are in some way connected with [sport] ^1^.*								
**Self-expression**		0.56	0.79	0.23–0.40		0.59	0.81	0.17–0.42
*When I participate in [sport] ^1^ I can really be myself.*								
You can tell a lot about a person be seeing them [sport] ^1^.	0.61				0.64			
When I participate in [sport] ^1^ other see me the way I want them to see me.	0.70				0.75			
[sport] ^1^ says a lot about who I am.	0.90				0.90			

^1^ running for running participants and walking for walking participants; items in italics were excluded from the analysis to improve the fit of the models; β: factor loading; AVE: average variance extracted; CR: composite reliability; SC: squared correlations between constructs (range).

**Table 2:** Final exploratory factor analyses of the scale to measure the motivation of the use applications and sports watches among running and walking participants.

Item ^1^	Running Participants—App					Walking Participants—App					Running Participants—Watch					Walking Participants—Watch				
	F1	F2	F3	F4	F5	F1	F2	F3	F4	F5	F1	F2	F3	F4	F5	F1	F2	F3	F4	F5
EE1				**0.61**					**0.78**					**0.66**					**0.64**	0.44
EE2				**0.71**					**0.65**	0.34				**0.65**					**0.71**	
EE3				**0.54**						0.75				**0.63**					**0.73**	
EE4				**0.62**			0.34		**0.47**					**0.53**					**0.55**	0.37
FC1			**0.59**					**0.64**					**0.57**					**0.67**		
FC2			**0.79**					**0.77**					**0.80**					**0.78**		
FC3			**0.80**					**0.76**					**0.73**					**0.76**		
HA1					**0.77**	0.56				**0.35**					**0.65**	0.40	0.41			**0.42**
HA2					**0.71**	0.35		0.38		**0.49**					**0.77**					**0.74**
HA3				0.36	**0.68**				0.41	**0.51**					**0.74**			0.42		**0.55**
HE1	**0.63**						**0.50**			0.45	**0.62**					**0.48**			0.36	
HE2	**0.88**						**0.85**				**0.86**					**0.87**				
HE4	**0.84**						**0.84**				**0.83**					**0.85**				
ID1		**0.68**				**0.71**						**0.70**					**0.76**			
PE1	**0.70**						**0.77**				**0.77**					**0.78**				
PE3	**0.61**						**0.68**				**0.57**		0.33			**0.72**				
PE5	**0.83**						**0.84**				**0.84**					**0.85**				
PV2			**0.62**					**0.72**					**0.62**					**0.68**		
PV3			**0.69**					**0.77**					**0.63**					**0.63**		
SI1		**0.77**				**0.66**						**0.65**					**0.66**			
SI2		**0.62**				**0.62**						**0.63**					**0.69**			
SI4		**0.82**				**0.76**						**0.77**		0.32			**0.72**			0.31
SI5		**0.82**				**0.78**						**0.77**					**0.74**			
SI6		**0.77**				**0.78**						**0.79**					**0.81**			
EV	7.0	2.5	2.0	1.6	1.3	8.2	2.3	1.8	1.3	1.0	6.8	2.6	2.0	1.3	1.2	7.8	2.8	2.1	1.2	1.0
% of var	29.3	10.3	8.5	6.6	5.4	34.3	9.7	7.6	5.6	4.4	28.5	10.8	8.2	5.5	5.0	32.6	11.8	8.9	4.9	4.1

F1: Enjoyment and performance expectancy; F2: Social influence; F3: Price and support values; F4: Effort expectancy; F5: Habit; EV: Eigenvalue; Items in bold indicate which factor each item belongs to; ^1^ For an overview of the individual items, see Table S2 (added as Supplementary Materials).

**Table 3:** Reliability of scale to measure motivation to use applications and sports watches among running and walking participants.

	Running Participants—App					Walking Participants—App					Running Participants—Watch					Walking Participants—Watch				
	F1	F2	F3	F4	F5	F1	F2	F3	F4	F5	F1	F2	F3	F4	F5	F1	F2	F3	F4	F5
Label	Enjoyment and performance expectancy	Social influence	Price and support values	Effort expectancy	Habit	Enjoyment and performance expectancy	Social influence	Price and support values	Effort expectancy	Habit	Enjoyment and performance expectancy	Social influence	Price and support values	Effort expectancy	Habit	Enjoyment and performance expectancy	Social influence	Price and support values	Effort expectancy	Habit
Items	6	6	5	4	3	6	6	5	4	3	6	6	5	4	3	6	6	5	4	3
Cronbach’s Alpha	0.88	0.87	0.80	0.59	0.72	0.89	0.85	0.85	0.57	0.67	0.88	0.84	0.78	0.64	0.66	0.90	0.84	0.83	0.72	0.67

**Table 4:** Descriptive statistics of running and walking participants, tested with chi-square tests and Bonferroni adjustment (in percentages).

Variable	Running Participants (N_weighted_ = 2146)	Walking Participants (N_weighted_ = 2767)	Sign.
**Sex**			***
Male	51.8 ^a^	45.0 ^b^	
Female	48.2 ^a^	55.0 ^b^	
**Age**			***
18–34 years old	30.7 ^a^	7.9 ^b^	
35–54 years old	58.2 ^a^	31.6 ^b^	
55 years old and older	11.1 ^a^	60.5 ^b^	
**Highest education achieved**			***
Lower and secondary education	43.0 ^a^	63.6 ^b^	
Higher education	57.0 ^a^	36.4 ^b^	
**General sports participation**			***
1–2 times/week	12.5 ^a^	32.0 ^b^	
3–4 times/week	53.5 ^a^	38.8 ^b^	
5 times/week or more	34.1 ^a^	29.2 ^b^	
**Specific running or walking participation**			***
1–2 times/week	34.4 ^a^	41.3 ^b^	
3–4 times/week	53.5 ^a^	31.2 ^b^	
5 times/week or more	12.1 ^a^	27.5 ^b^	
**Main sport**			**
Running or walking	76.9 ^a^	73.3 ^b^	
Other sport	23.1 ^a^	26.7 ^b^	
**Event participation**			***
Never	13.6 ^a^	23.3 ^b^	
At least once	86.4 ^a^	76.7 ^b^	
**Wearable usage ^1^**			***
Yes	91.4 ^a^	59.5 ^b^	
No	8.6 ^a^	40.5 ^b^	
**Type of wearable used**			
Application for smartphone	42.4 ^a^	65.9 ^b^	***
(Sports)watch/smartwatch (very suitable during sport)	84.8 ^a^	45.6 ^b^	***
Activity tracker (less suitable during sport)	5.0 ^a^	9.7 ^b^	***
Handheld GPS	2.0 ^a^	13.8 ^b^	***
**Most important type of wearable used**			***
Application for smartphone	18.8 ^a^	48.6 ^b^	
(Sports)watch/smartwatch (very suitable during sport)	79.6 ^a^	38.7 ^b^	
Activity tracker (less suitable during sport)	1.6 ^a^	6.0 ^b^	
Handheld GPS	0.1 ^a^	6.7 ^b^	
**Number of wearables used**			NS
Only one wearable	68.5 ^a^	69.4 ^a^	
Two or more wearables	31.5 ^a^	30.6 ^a^	
**What do you register with wearable ^2^**			
Running time	97.3 ^a^	80.1 ^b^	***
Distance	97.3 ^a^	89.8 ^b^	***
Speed	91.7 ^a^	51.6 ^b^	***
Heart rate	78.9 ^a^	34.5 ^b^	***
Cadence	49.3 ^a^	13.2 ^b^	***
Route	79.2 ^a^	56.4 ^b^	***
**What do you do with your registered data ^2^**			
I do not do anything with this data	3.1 ^a^	19.9 ^b^	***
I look at this data after my training	91.1 ^a^	72.1 ^b^	***
I use this data to monitor my progress	63.2 ^a^	17.0 ^b^	***
I use this data to adjust my workouts	22.5 ^a^	3.4 ^b^	***

^1^ for example: application for smartphone, (sports)watch/smartwatch, activity tracker, handheld GPS; ^2^ relates to most important type of wearable; *** *p* < 0.001; ** *p* < 0.01; ^a,b^ differ significantly; NS: not significant.

**Table 5:** Binary logistic regression for the most important wearable among running participants: use of applications (=0) or (sports)watches/smartwatches (=1) (N_weighted_ = 2050).

Variable	Model 1 Exp(B)	Model 2 Exp(B)	Model 3 Exp(B)	Model 4 Exp(B)	Model 5 Exp(B)	Model 6 Exp(B)
**Sex (ref. = male)**						
Female	0.651 ***	0.648 ***	0.646 ***	0.646 **	0.746	0.704
**Age (ref. = 18–34 years old)**						
35–54 years old	1.648 ***	1.429 **	1.413 *	1.295	1.283	1.169
55 years old and older	1.870 **	1.347	1.302	1.342	1.283	1.117
**Education (ref. = primary/secondary education)**						
Higher education	0.866	0.940	0.954	1.003	1.004	0.982
**Training frequency (ref. = 1–2 times/week)**						
3–4 times/week		1.646 ***	1.656 ***	1.522 **	1.492 *	1.541 *
5 times/week or more		2.286 **	2.267 **	2.096 **	2.505 **	2.765 **
**Main sport (ref. = running)**						
Other sport		1.281	1.298	1.354	1.255	1.292
**Event participation (ref. = never)**						
At least once		2.041 ***	2.035 ***	2.367 ***	2.230 ***	2.151 ***
**Attraction (involvement)**		1.332 *	1.321 *	1.303 *	1.431 *	1.428 *
**Centrality (involvement)**		1.711 ***	1.738 ***	1.670 ***	1.576 ***	1.554 **
**Self-expression (involvement)**		0.862	0.858	0.871	0.810	0.817
**Number of wearables used (ref. = only one)**						
Multiple			0.823	0.738 *	0.476 ***	0.482 ***
**Enjoyment and performance expectancy (motivation)**				1.058	0.966	1.018
**Social influence (motivation)**				0.561 ***	0.577 ***	0.568 ***
**Price and support values (motivation)**				2.136 ***	1.351 *	1.324
**Effort expectancy (motivation)**				1.107	1.089	1.178
**Habit (motivation)**				1.604 ***	1.546 ***	1.602 ***
**Registered with wearable** **(ref. = not registered)**						
Running time					1.796	2.046
Distance					0.651	0.565
Speed					1.043	1.133
Heart rate					32.310 ***	36.364 ***
Cadence					1.690 *	1.792 **
Route					0.348 ***	0.345 ***
**Purpose of collected data (ref. = not checked)**						
I do not do anything with this data						0.861
I look at this data after my training						1.071
I use this data to monitor my progress						0.519 **
I use this data to adjust my workouts						0.936
Nagelkerke R²	0.033	0.166	0.168	0.266	0.586	0.592
Model χ² (df)	39.312 (4) ***	207.969 (11) ***	210.085 (12) ***	343.966 (17) ***	864.437 (23) ***	875.502 (27) ***

Dependent variable: use of applications = 0/use of (sports)watches or smartwatches = 1; *** *p* < 0.001; ** *p* < 0.01; * *p* < 0.05.

**Table 6:** Binary logistic regression for the most important wearable among walking participants: use of applications (=0) or (sports)watches/smartwatches (=1) (N_weighted_ = 1628).

Variable	Model 1 Exp(B)	Model 2 Exp(B)	Model 3 Exp(B)	Model 4 Exp(B)	Model 5 Exp(B)	Model 6 Exp(B)
**Sex (ref. = male)**						
Female	1.115	1.136	1.228	1.337 *	1.211	1.171
**Age (ref. = 18–34 years old)**						
35–54 years old	0.661 *	0.699	0.690	0.751	0.730	0.731
55 years old and older	0.425 ***	0.450 ***	0.492 **	0.528 **	0.544 *	0.535 *
**Education (ref. = primary/secondary education)**						
Higher education	1.045	0.971	0.947	1.008	1.083	1.097
**Training frequency (ref. = 1–2 times/week)**						
3–4 times/week		1.373 *	1.300	1.214	1.256	1.298
5 times/week or more		1.350 *	1.374 *	1.123	0.956	0.997
**Main sport (ref. = running)**						
Other sport		1.667 ***	1.690 ***	1.517 **	1.216	1.208
**Event participation (ref. = never)**						
At least once		1.079	0.981	1.019	1.018	1.019
**Attraction (involvement)**		1.119	1.154	0.950	0.929	0.960
**Centrality (involvement)**		0.934	0.930	0.907	0.944	0.940
**Self-expression (involvement)**		0.890	0.870	0.863	0.982	0.961
**Number of wearables used (ref. = only one)**						
Multiple			2.619 ***	2.442 ***	1.610 **	1.669 **
**Enjoyment and performance expectancy (motivation)**				1.095	0.943	0.962
**Social influence (motivation)**				0.476 ***	0.504 ***	0.511 ***
**Price and support values (motivation)**				1.757 ***	1.439 **	1.465 **
**Effort expectancy (motivation)**				0.998	0.826	0.835
**Habit (motivation)**				1.886 ***	2.017 ***	2.016 ***
**Registered with wearable** **(ref. = not registered)**						
Running time					0.852	0.850
Distance					1.053	1.074
Speed					0.689	0.689
Heart rate					30.553 ***	31.773 ***
Cadence					1.167	1.168
Route					0.712	0.707
**Purpose of collected data (ref. = not checked)**						
I do not do anything with this data						2.587 *
I look at this data after my training						2.270 *
I use this data to monitor my progress						0.949
I use this data to adjust my workouts						0.727
Nagelkerke R²	0.029	0.053	0.110	0.248	0.576	0.581
Model χ² (df)	30.901 (4) ***	56.466 (11) ***	119.319 (12) ***	284.889 (17) ***	781.599 (23) ***	791.099 (27) ***

Dependent variable: use of applications = 0/use of (sports) watches or smartwatches = 1; *** *p* < 0.001; ** *p* < 0.01; * *p* < 0.05.

## Data Availability

The data presented in this study are available from the corresponding author, upon reasonable request.

## Supplementary Materials

The following supporting information can be downloaded at: https://www.mdpi.com/article/10.3390/ijerph19159284/s1, Table S1: Exploratory factor analyses of the scale to measure motivation the use applications and (sports)watches/smartwatches among running and walking participants; Table S2: Overview of final factor structure of the scale to measure motivation to use applications and (sports)watches/smartwatches among running and walking participants.
