# Supplementary material for: Two of a Kind? Similarities and Differences between Runners and Walkers in Sociodemographic Characteristics, Sports Related Characteristics and Wearable Usage
Source: Int J Environ Res Public Health. 2022 Jul 29;19(15):9284. doi: 10.3390/ijerph19159284 (PMC9368676; doi:10.3390/ijerph19159284)
Supplement: Supplementary file 1 [file ijerph-19-09284-s001.zip › ijerph-1797948 - Table S1.pdf]

International Journal of
Environmental Research and Public Health

MDPI

Table S1. Exploratory factor analyses of the scale to measure motivation the use applications and (sports)watches/smartwatches among running and walking participants.

Item	Running participants (app)						Walking participants (app)						Running participants (watch)					Walking participants (watch)				
	F1	F2	F3	F4	F5	F6	F1	F2	F3	F4	F5	F6	F1	F2	F3	F4	F5	F1	F2	F3	F4	F5
EE1				0.62						0.56	0.54					0.66					0.77	
EE2				0.69						0.76						0.59					0.64	
EE3				0.52								0.82				0.57					0.65	
EE4				0.61			0.32				0.61					0.52					0.58	0.39
FC1			0.59						0.64						0.57					0.65		
FC2			0.70			0.42			0.76						0.78					0.77		
FC3			0.71			0.34			0.75						0.72					0.75		
HA1					0.76		0.32	0.62									0.65	0.439	0.51			
HA2			0.31		0.69			0.38	0.39	0.35		0.32					0.75				0.45	
HA3				0.37	0.65					0.62							0.72			0.44	0.48	
HE1	0.62						0.49					0.50	0.63					0.45			0.33	
HE2	0.87						0.84						0.85					0.86				
HE4	0.84						0.84						0.82					0.85				
ID1		0.70						0.74						0.71					0.78			
ID3		*0.49*			*0.31*			*0.53*						*0.51*					*0.53*			
PE1	0.70						0.78						0.78					0.77				
PE2	*0.35*				*0.34*	*0.45*	*0.46*						*0.42*			*0.35*		*0.50*				*0.41*
PE3	0.61						0.69						0.57		0.32			0.72				
PE5	0.83						0.83						0.84					0.84				
PE6						*0.70*	*0.36*				*0.39*		*0.39*							*0.31*		*0.65*
PV2			0.68						0.71						0.62					0.68	0.30	
PV3			0.77						0.76						0.63					0.62		
SI1		0.79						0.66						0.66					0.64			
SI2		0.63						0.61						0.64					0.69			
SI4		0.80						0.71			0.36			0.75		0.37			0.72			
SI5		0.80						0.72			0.33			0.75		0.33			0.75			
SI6		0.78						0.77						0.77					0.79			
EV	7.7	2.6	2.1	1.6	1.4	1.1	9.1	2.4	1.8	1.4	1.0	1.0	7.5	2.7	2.0	1.4	1.2	8.7	2.9	2.2	1.2	1.0
% of var	28.4	9.8	7.7	6.0	5.0	4.2	33.5	8.9	6.8	5.0	3.9	3.7	27.6	10.2	7.3	5.1	4.6	32.2	10.9	8.1	4.4	3.9

Items in *italics* were excluded from the analysis.
