# Supplementary material for: Two of a Kind? Similarities and Differences between Runners and Walkers in Sociodemographic Characteristics, Sports Related Characteristics and Wearable Usage
Source: Int J Environ Res Public Health. 2022 Jul 29;19(15):9284. doi: 10.3390/ijerph19159284 (PMC9368676; doi:10.3390/ijerph19159284)
Supplement: Supplementary file 1 [file ijerph-19-09284-s001.zip › ijerph-1797948 - Table S2.pdf]

International Journal of
Environmental Research and Public Health

MDPI

Table S2. Overview of final factor structure of the scale to measure motivation to use applications and (sports)watches/smartwatches among running and walking participants.

Variable (I use my monitoring device because …)	Label	Factor
the [wearable] increases my feelings of joy during running[1]	HE1	Enjoyment and performance expectancy
the [wearable] motivates me to run[1]	HE2	
the [wearable] makes me run[1] more often	HE4	
my experiences with the [wearable] influenced my running[1] behavior	PE1	
the [wearable] contributes to my health	PE3	
it makes me more motivated to run[1]	PE5	
runners[1] in my environment expect me to use the [wearable]	ID1	Social influence
runners[1] in my environment also use the [wearable]	SI1	
runners[1] recommended the [wearable]	SI2	
I can share my training with people in my environment	SI4	
the [wearable] allows people in my environment to follow me	SI5	
I can connect online with other runners[1] with the [wearable]	SI6	
the [wearable] has good online support	FC1	Price and support values
I can set up the [wearable] according to my preferences	FC2	
this supports me in using the [wearable]	FC3	
the [wearable] has a good price-quality ratio	PV2	
the benefits of the [wearable] outweigh the costs occurred in time and effort	PV3	
the [wearable] easily tracks my running[1] performance	EE1	Effort expectancy
the [wearable] is easy to use	EE2	
the [wearable] displays the correct information	EE3	
I can look back at training sessions with the [wearable]	EE4	
I can't run[1] without the [wearable]	HA1	Habit
it is part of my running[1] equipment	HA2	
it is a habit to run[1] with the [wearable]	HA3	

[1] walking/walk/walker for walking participants.
